# Emergency Department Non-Urgent Visits and Hospital Readmissions Are Associated with Different Socio-Economic Variables in Italy

**DOI:** 10.1371/journal.pone.0127823

**Published:** 2015-06-15

**Authors:** Pamela Barbadoro, Elena Di Tondo, Vincenzo Giannicola Menditto, Lucia Pennacchietti, Februa Regnicoli, Francesco Di Stanislao, Marcello Mario D’Errico, Emilia Prospero

**Affiliations:** 1 Department of Biomedical Science and Public Health, Università Politecnica delle Marche, Ancona, Italy; 2 Hospital Hygiene Service, AOU Ospedali Riuniti, Ancona, Italy; 3 Department of Emergency Care, AOU Ospedali Riuniti, Ancona, Italy; Azienda Ospedaliero-Universitaria Careggi, ITALY

## Abstract

**Objective:**

The aim of this paper was to evaluate socio-economic factors associated to poor primary care utilization by studying two specific subjects: the hospital readmission rate, and the use of the Emergency Department (ED) for non-urgent visits.

**Methods:**

The study was carried out by the analysis of administrative database for hospital readmission and with a specific survey for non-urgent ED use.

**Results:**

Among the 416,698 sampled admissions, 6.39% (95% CI, 6.32–6.47) of re-admissions have been registered; the distribution shows a high frequency of events in the age 65–84 years group, and in the intermediate care hospitals (51.97%; 95%CI 51.37–52.57). The regression model has shown the significant role played by age, type of structure (geriatric acute care), and deprivation index of the area of residence on the readmission, however, after adjusting for the intensity of primary care, the role of deprivation was no more significant. Non-urgent ED visits accounted for the 12.10%, (95%CI 9.38–15.27) of the total number of respondents to the questionnaire (N = 504). The likelihood of performing a non-urgent ED visit was higher among patients aged <65 years (OR 3.2, 95%CI 1.3–7.8 p = 0.008), while it was lower among those perceiving as urgent their health problem (OR 0.50, 95%CI 0.30–0.90).

**Conclusions:**

In the Italian context repeated readmissions and ED utilization are linked to different trajectories, besides the increasing age and comorbidity of patients are the factors that are related to repeated admissions, the self-perceived trust in diagnostic technologies is an important risk factor in determining ED visits. Better use of public national health care service is mandatory, since its correct utilization is associated to increasing equity and better health care utilization.

## Introduction

The implementation of a universal, publicly-funded national health care service in Italy was justified on the hypothesis that all citizens should have had access to health-care services on the basis of their need rather than of their ability to pay. Equity of access is a key element of health system performance in OECD countries [1,2]. However, recent papers have demonstrated that significant inequities in primary healthcare do exists in selected OECD countries [3, 4]. In the context of healthcare system evaluation, hospital readmissions are usually considered as markers of costly, suboptimal healthcare in the primary setting [5,6], and have also negative social impacts [7]. Strong link between income inequality and readmission risk has been recently demonstrated [8, 9]. In addition, some studies have shown that hospitalization rates are lower where a better primary care access can be found [10], and that 30 days readmission rates for some conditions (pneumonia, heart attack, and heart failure) decrease as the number of family physician increases [11]. On the other hand, another potential indicator of difficulties in primary health care is the use of Emergency Department (ED) services for non-urgent care [12, 13, 14]. Across Europe, different models of after-hours primary care exist [15, 16]; however, despite good primary care access, high and rising ED visits still represent an issue [17, 18]. Emergency primary care in Italy currently consist of a combination of volunteer organizations providing ambulance service, supplemented by physicians and nurses who perform advanced life support (ALS) procedures. People seeking voluntary emergency primary care, may refer to the hospital Emergency Department free of charge; a small amount maybe charged in case of non-urgent visits. Moreover, people not perceiving their medical problem as urgent, may refer free of charge to their General Practitioner, during the day, and to the emergency medical service, during the night and weekends.

The main objectives of the study have been to analyze the variables associated to: a) hospital readmissions and b) to ED utilization for non-urgent care in the area of interest.

## Methods

Written informed consent was required for the collection and processing of sensitive data. The survey was approved by the local Ethics Committee of the Ospedali Riuniti of Ancona, Italy, were the study was performed. Informed consent was obtained from all study participants. Hospital admission data did not required a second approval by local ethics committee since data had been previously anonymized and de-identified by Professionals belonging the Regional Health Information System (which is responsible for health care data management in the Marches Region) prior to access and analysis. The study was carried out in the Marches, a region situated in Central Italy, in the 2008–2011 period.

At the time of the survey the primary health-care delivery in the Marches region was provided by 24 local health districts (LHD), with a discrete level of autonomy in delivering primary care services. The LHDs form the basic elements of the Italian NHS; they provide free primary care, home care as well as residential/intermediate care, and rehabilitation, integrated with social services (provided by the local municipalities administration). Primary health care is provided mainly by General Practitioners (GPs), and on-call physicians for afterhours medical care and services. The choice of primary care services (e.g.: home care/residential care) is targeted to patients after the multivariate assessment of patients clinical and socio-economic needs, made by a geriatric evaluating unit (including patients’ General Practitioner, a specialized nurse, specialized doctors, and a social worker).

In our study we have performed an analysis of some socio-economic factors to check the use of primary health care use. This analysis has been carried out by two main phases: a) the analysis of hospital readmission data in the area of interest, and b) the analysis of ED utilization for non-urgent care.

### Hospital readmission data

The 28-day readmission rate was defined as any repeated admission within 28 days after being discharged alive from an index hospital admission, divided by the total number of index admissions [19]. The 28 days limit has been chosen because of its inclusion amongst the Italian National Outcomes Program by the Italian Ministry of Health [20]. Besides the difficulties in defining an accurate indicator for hospital readmission [21], the 28 days deadline has been previously validated as a statistically significant deadline [22].

A sample of discharge data (2008–2009 years) belonging to all the hospitals in the Region have been included in the analysis. Subjects over the age of 18 years were included in the study, while day-hospital admissions, and trauma events were excluded. The data set allowed patients to have more than one re-admission episode, but each re-admission within 28days was linked only with the most recent prior admission. Early discharge was defined as discharge occurring before the national trimpoint of length of stay for each Diagnosis Related Group (DRG). According to a decree by the Italian Ministry of Health dating back to 2008, a trimpoint of LOS, specific for each DRG, has been introduced, in order to identify admissions with an unusual LOS (i.e. trimpoint for DRG n. 87 Pulmonary Edema and Respiratory Failure is of 27 days).

From the analysis of hospital discharge records, variables describing the episode of hospitalization were detected: type of admission (Geriatric acute care/others), type of discharge (early, other), and Major Diagnostic Category (MDC), the main and secondary diagnoses (classified according to the ICD9-CM), and internal transfers.

For the evaluation of variables associated to repeated admissions, information about demographic characteristics (age, sex, marital status), and residence (home or nursing facility) have been included at an individual level; moreover, at an organizational/environmental level, information about deprivation of the living area, and organization of primary health care delivery (number of GPs/1,000 inhabitants, number of long-term acute care beds/1,000 inhabitants, number of elderly patients assisted in home care/1,000 inhabitants >65 years old) have been evaluated.

For the assessment of the social capital of the patients’ area of residence, a small-area level index of socioeconomic deprivation previously validated in Italy has been used [23]; this Disadvantaged Areas Index (DAI) was calculated by using data from the 2001 census by town. The index is built on the following variables: proportion of primary school educated residents, proportion of unemployed, proportion of homes without bathroom, proportion of families living in rental housing, proportion of single parent families with dependent children. Index was calculated by summing the scores of the selected variables, which is the deviation from the regional average divided by the standard deviation of the entire region; thereafter, it was categorized into quintiles. Administrative hospital discharge data had been anonymized, and de-identified by Professionals belonging the Regional Health Information System prior to access and analysis by researchers, therefore its analysis did not require any evaluation by the local ethics committee.

### Non-urgent Emergency Department visits

For the analysis of the non-urgent ED admissions, a sample of admissions of subjects aged ≥ 18 years was enrolled at the ED of the 900-bed teaching hospital of Ancona, the main hospital in Marches Region. Information about enrolled patients was acquired through an anonymous questionnaire (S1 Questionnaire).

All the following variables have been included in the questionnaire completed by the patient: a) socio-economic variables: gender, age class, marital status, education, occupation, type of family, living condition, distance from hospital to home and citizenship, b) variables related to the motivations of access to the ED: sense of urgency by the patient, financial difficulties, presence of recent traumatic injury, difficulty to contact the GP, greater confidence in the hospital, previous medical therapy without benefit, too long waiting times for booking health examinations, more tools to solve clinical problem in the ED, easy accessibility of ED, and c) variables related to clinical characteristics: presence of chronic diseases, Charlson Comorbidity Index score, presence of chronic ongoing drug therapy, self perceived health status, previous clinical examination in the past 12 months, previous access in the ED last year, ED arrival, attempting to contact the GP before arriving at the ED, and duration of the clinical problem.

The questionnaire has been validated for validity face, reliability and consistency, in a pilot phase involving 50 subjects. Face and content validity were established through the evaluation of the questionnaire’s items by experts in both general medicine, nursing, and public health. Concurrent construct validity was estimated by comparing specifically designed items within the instrument with other items measuring the same concepts.

The reliability coefficient for dichotomous variables (Kuder-Richardson test) was 0.95, and for Likert scale items (Cronbach’s alpha) was greater than 0.73.

In order to investigate the issue of inequalities in access to services of the foreign population in the questionnaire, the information letter and informed consent have been translated into several languages. Written informed consent was required for the collection and processing of sensitive data was obtained from all participants and was obtained from all participants. The survey was approved by the Ethics Committee of Ospedali Riuniti of Ancona, Italy, were the study was performed. The observation was carried out from 8 a.m. to 8 p.m. in working days in 2011.

According to previous published surveys [24, 25] the level of urgent care has been defined as:

1. Extreme emergency: the patient should be seen promptly by a physician in the ED to assess and treat possible life-threatening conditions, and immediate care was necessary within 24 h in order to avoid severe consequences for the patient;
2. Emergency: the patient required care within 24–48 h, or the technical equipment of the hospital had to be used for diagnosis or therapeutic purposes;
3. Emergency as perceived by the patient: the patient was worried by the appearance or the recent worsening of symptoms (e.g. a left arm pain or a chest pain, which could be related to an acute myocardial infarction), although the vital or functional prognosis was not threatened within 24 h. The patient’s condition is appropriate for referral to a general or subspecialty clinic;
4. Non urgent: the patient has no active symptoms or they were recent and minor, without any feeling of emergency and he/she desires a check-up, a prescription refill or a return-to-work release.

The definition of urgency of patient’s visit (i.e. urgent, non-urgent visit), according to the above methodology, has been based on clinical judgment made by clinicians during the medical examination at the ED.

### Statistical analysis

Univariate and bivariate analysis have been performed to describe the characteristics of readmissions and of the non-urgent ED visits. Chi squared test was used to assess significance of bivariate associations. Moreover, distinct methods have been used to analyze hospital readmissions and non-urgent care visits, as follows:

1. **Hospital readmission data.** For the analysis of hospital readmissions, a multilevel regression model was built to estimate factors associated to the hospital readmission, clustering observations at a local healthcare area level. This kind of analysis allows treating data in a hierarchical/nested structure, in which the observation units are grouped into larger units, such as local health districts of residence of patients. The hierarchical model was chosen to estimate the role of primary care, and social services in caring for patients. The following independent variables were selected and entered into the model: age (18–44 = 1, 45–64 = 2, 65–79 = 3, ≥80 = 4), presence of cardiac ischemia (1 = present, 0 = absent), presence of Chronic Renal Failure (CRF, 1 = present, 0 = absent), previous early discharge from hospital (1 = present, 0 = absent), hospitalization in Geriatric care facilities (1 = present, 0 = absent), living in residential care (1 = present, 0 = absent), deprivation index of living area (five quintiles: ranging between 1 as the less deprived areas, and 5 as the most deprived).The significance level for variables to enter the multiple logistic regression models was set at <0.20, and for removing them from the model at >0.40; socio-demographic, clinically and organizationally meaningful variables have been, however, included in the final analysis. Intraclass correlation for pairs of latent linear responses at each nested level of the model. The accuracy of the logistic model in predicting the possibility of future readmission was assessed by receiver operating characteristic (ROC) analysis using the DeLong nonparametric method.
2. **Non-urgent Emergency Department visits.** For the analysis of ED non urgent visits characteristics multiple logistic regression models were developed to adjust for confounding, and to evaluate which factors were independently associated with non urgent admission. ED visits have been studied in the ED of the main, tertiary care, hospital in Marches Region. The independent variables included in the regression models were classified as follows:a) socio-demographic variables: gender (1 = male; 2 = female), age classes (1 = 18–65 years; 0 = older than 65 years), marital status (1 = married/cohabitant; 2 = single; 3 = missing), education (1 = no qualification/primary school/middle school; 2 = high school/undergraduate and postgraduate; 3 = missing), occupation (1 = student, 2 = unemployed, 3 = unable to work, 4 = housewife, 5 = manager/freelancer, 6 = craftsman/merchant/technician, 7 = employee/teacher, 8 = workman, 9 = missing), type of family (1 = living alone 2 = living with partner/relatives, 3 = other, such as public home, or homeless, 4 = missing), living condition (1 = home ownership/rent, 2 = community, 3 = other, 4 = missing), distance from hospital to home (1 = less than 5 km, 2 = 5–35 km, 3 = more than 35 km, 4 = missing) and citizenship (1 = Italian, 2 = foreign, 3 = missing);b) variables related to the motivations of access to the ED: sense of urgency by the patient (1 = present, 0 = absent), financial difficulties (1 = present, 0 = absent), presence of recent traumatic injury (1 = present, 0 = absent), difficulty to contact the GP (1 = present, 0 = absent), greater confidence in the hospital (1 = present, 0 = absent), previous medical therapy without benefit (1 = if present, 0 = absent), too long waiting times for booking exams (1 = present, 0 = absent), more tools to solve clinical problem in the ED (1 = present, 0 = absent), easy accessibility of ED (1 = present, 0 = absent);c) variables related to clinical characteristics: presence of chronic diseases (0 = absent,1 = present, 2 = missing), Charlson Index score (1 = 0 points, 2 = score between 1 and 2 points, 3 = score between 3 and 4 points and 4 = score ≥5 points), presence of chronic ongoing drug therapy (0 = absent, 1 = present, 2 = missing), self perceived health status (1 = very good, 2 = good, 3 = moderate, 4 = poor, 5 = very poor, 6 = missing), previous clinical examination in the past 12 months (0 = absent, 1 = present, 2 = missing), previous access in the ED last year (0 = absent, 1 = present, 2 = missing), ED arrival (1 = independent decision/by suggestion of relatives or friends, 2 = on medical advice, 3 = missing), attempting to contact the GP before arriving at the ED (0 = absent, 1 = present, 2 = missing) and duration of the clinical problem (1 = one hour, 2 = less than one day, 3 = more than one day, 4 = missing).The significance level for variables to enter the multiple logistic regression models was set at ≤0.20, and for removing them from the model at ≤0.40.

Data were collected using Microsoft Access, Stata software package 9.0 was used for statistical analysis (Stata Corp., College Station, TX, 2007). The level of significance was set at 0.05.

## Results

### Hospital readmission data

Among the 416,698 sampled admissions, a total of 26,627 readmissions have been registered in the observation period, corresponding to an overall 6.39% (95% CI, 6.32–6.47) readmission rate (Table 1).

**Table 1:** Distribution of repeated readmissions according to clinical and organizational characteristics, Marches region.

Variable		Repeated hospitalization rate		^a^p-Value
		N	%	
Gender	Male	15,495	7.04	*
	Female	11,132	5.12	
Age class	18–44	3,118	3.99	*
	45–64	5,881	6.60	
	65–79	10,921	7.05	
	≥80	6,707	5.82	
Marital status	Married	14,178	6.55	*
	Separated/Divorced	768	5.37	
	Widowed	2,413	6.79	
	Single	3,289	5.87	
	Not known	5,979	5.25	
Type of hospital	Geriatric acute care	2,382	7.93	
	Other	24,245	5.95	
Renal failure	Present	1,412	8.26	
	Absent	25,215	6.00	
Ischemia	Present	2,515	6.92	*
	Absent	24,112	6.01	
Diabetes	Present	3,941	7.67	
	Absent	22,686	5.88	
Hypertension	Present	4,374	5.38	
	Absent	22,253	6.25	
Number of previous admissions	3 or more	18,545	12.80	*
	Less than 3	8,082	2.76	
Living town deprivation index	1 (most affluent)	10,798	7.39	*
	2	5,701	5.40	
	3	4,814	5.03	
	4	4,567	6.06	
	5 (most deprived)	756	5.09	
Living	Home	212	5.67	
	Nursing home	26,415	6.09	

^a^p-Value

*<0.05

Male patients seem to be at higher risk of repeated hospitalization with respect to women, with 58.19% of total readmissions, and a readmission rate of 7.04% versus 5.12% (p<0.05).The distribution shows a high frequency of events in the age group 65–79 years (rate 7.05%), with a lower impact in admissions in the most advanced age group (rate 5.82, p<0.05).

The distribution of admissions by type of structure highlights the burden of care on geriatric hospitals which account for 51.97% of hospital readmissions, with a frequency of 7.93% versus the 5.95% found in other types of structures (p<0.05).

The multilevel regression (Table 2) has shown the significant role played by age on the readmission event (OR 1.67, 95%CI1.40–2.00 between 45 and 64 years, OR 1.81, 95%CI1.53–2.13 between 65 and 74 years, OR 1.72, 95%CI 1.44–2.05 for patients aged over 85), while reducing the significance of patients’ sex to a non-significant variable. The type of structure is important too, and readmissions were common in geriatric facilities (OR 2.83, 95%CI2.35–3.40).

**Table 2:** Comparison of variables associated with repeated hospitalization, at multivariable modeling and mixed effect multilevel modeling with primary care health authority as clustering variable.

Variable		a. Without primary care level			b. With primary care level		
		OR	95%CI	^a^p-Value	OR	95%CI	^a^p-Value
Age groups (year)							
	18–44	1			1		
	45–64	1.67	1.40–2.00	*	1.67	1.40–2.00	*
	65–79	1.83	1.55–2.16	*	1.81	1.53–2.13	*
	≥80	1.81	1.52–2.16	*	1.72	1.44–2.05	*
Type of facility							
	Geriatric acute care	2.24	1.84–2.73	*	2.83	2.35–3.40	*
MDC								
	Ischemic heart disease	1.37	1.13–1.68	*	1.42	1.16–1.74	*
	Chronic renal failure	1.50	1.17–1.94	*	1.51	1.17–1.95	*
Previous hospital utilization							
	Number of previous admissions	2.10	2.04–2.17	*	2.11	2.05–2.18	*
	Earlydischarge	2.93	2.31–3.73	*	2.86	2.24–3.63	*
Living town deprivation index							
	1 (most affluent)	1			1		
	2	1.81	1.53–2.16	*	1.69	1.15–2.49	*
	3	1.21	1.01–1.45	*	1.11	0.77–1.61	
	4	1.25	1.04–1.50	*	1.34	0.92–1.94	
	5 (most deprived)	1.55	1.28–1.88	*	1.47	0.99–2.18	
Living							
	Home	1			1		
	Nursing home	1.82	1.15–2.89	*	1.72	1.08–2.74	*

Ischemic heart disease (OR 1.42, 95%CI1.16–1.74), and chronic renal failure (OR 1.51, 95%CI1.17–1.95) were the most common disease in readmissions. Moreover readmissions are more common within patients with a high number of hospitalization in the previous year (OR 2.11, 95%CI2.05–2.18), and in cases with previous early hospital discharge (OR 2.86, 95%CI2.24–3.64).

The multilevel regression model has highlighted the significant role played by the local health authority of residence, in reducing the potential role played by the deprivation index (Table 2). In fact, the role of the local health authority of residence (estimated from the infraclass correlation coefficient), accounted for about 20.32% (95%CI 12.60–32.77%) of the variability observed in the sample.

### Non-urgent visits to the Emergency Department

In addition, 504 interviews have been collected in patients admitted to the ED; the response rate to the questionnaire was of 29.0%, response rate was slightly higher in non-urgent (36.5%), versus urgent patients’ (28.2%), p<0.05. 270 of participants (53.57%, 95%CI 49.10–57.99) were male, with a mean age of 47.9 years (DS 20.7 years). About a half of the patients enrolled in the survey had a high level of education (48.41%, 95%CI 43.97–52.87); 82.94% (95%IC 79.36–86.11) of participants lived with the family. Concerning the job, 23.81% (95%CI 20.15–27.77) was employed. Half of the patients did not suffer from any chronic diseases, and did not take any medication (Table 3).

**Table 3:** Distribution of characteristics of patients interviewed at the ED, and of urgency of their visit.

Variable		Total		Non urgent (Tot 61)		^a^p-Value
		N	%	N	%	
Gender	Male	270	53.57	33	12.22	
	Female	234	46.43	28	11.97	
Age class	18–65 years	366	72.62	52	14.21	*
	≥65 years	138	27.38	9	6.52	
Marital status	Married/cohabitant	280	55.56	38	13.57	
	Single	214	42.46	21	9.81	
	Missing	10	1.98	2	20.00	
Education	No qualification/Primary school/Middle school	252	50.00	25	9.92	
	High school/Undergraduate and postgraduate	244	48.41	35	14.34	
	Missing	8	1.59	1	12.50	
Occupation	Student	53	10.52	12	22.64	
	Unemployed	27	5.36	3	11.11	
	Unable to work	7	1.39	2	28.57	
	Housewife	76	15.08	8	10.53	
	Manager/Freelancer	34	6.75	2	5.88	
	Craftsman/Merchant/Technician	53	10.52	5	9.43	
	Employee/Teacher	80	15.87	9	11.25	
	Workman	120	23.81	17	14.17	
	Missing	54	10.71	3	5.56	
Type of family	Living alone	61	12.10	2	3.28	
	Living with a partner/relatives	418	82.94	56	13.40	
	Other	17	3.37	3	17.65	
	Missing	8	1.59	0	0.00	
Where lives	Home ownership/rent	487	96.63	60	12.32	
	Community	1	0.20	0	0.00	
	Other	11	2.18	0	0.00	
	Missing	5	0.99	1	20.00	
Distance from hospital to home	<5 km	132	26.19	15	11.36	
	5–35 km	325	64.48	41	12.62	
	>35 km	37	7.34	3	8.11	
	Missing	10	1.98	2	20.00	
Cityzenship	Italian	438	86.90	52	11.87	
	Foreign	63	12.50	9	14.29	
	Missing	3	0.60	0	0.00	

^a^p-Value

*<0.05

Analysis of the characteristic of the ED visits showed that the admission referred as “non-urgent” accounted for 12.10% (95%CI 9.38–15.27) of the total. 58.33% (95%CI 53.89–62.67) of patients decided to go to the ED on the basis of an independent decision (self-referral) or by the suggestion of relatives or friends, and 54.96% (95%CI 50.49–59.36) did not consult their General Practitioner (GP) before going the ED, although 85.5% (95%CI 82.4–88.6) of respondents declared to know their GPs opening hours. Bivariate analysis revealed that age-related differences were statistically significant: among non-urgent visits, age group <65 years was the most represented (14.21%, p<0.05). In addition 6.57% of patients with chronic disease and 5.52% of patients that take on a therapy has a non-urgent ED visit (p<0.05) and bivariate analyses showed also that the proportion of non-urgent visits raises by increasing the duration of the clinical problem from one hour to more than a day (from 1.12% to 20.53%, p<0.05) (Table 4 and Table 5). Logistic regression partially confirmed the results of bivariate analysis revealing that the likelihood of performing a non-urgent ED visit was higher among patients aged <65 years (OR 3.2, 95%CI 1.3–7.8), and was lower among those perceiving as urgent their health problem (OR 0.50, 95%CI 0.30–0.90) and among them who present their clinical problem longer than a day (OR 30.7, 95%CI 4.0–223.7).

**Table 4:** Distribution of non urgent ED visits according to the motivation of access to ED.

Variable		Total		Non urgent (tot 61)		^A^p-value
		N	%	N	%	
Personal reasons						
Urgency perceived by the patient	Present	235	46,63	23	9,79	
	Absent	269	53,37	38	14,13	
Financial difficulties	Present	9	1,79	0	0,00	
	Absent	495	98,21	61	12,32	
Recent traumatic injury	Present	179	35,52	14	7,82	*
	Absent	325	64,48	47	14,46	
Reasons relating to the GP						
Difficulty to contact the GP	Present	66	13,10	9	13,64	
	Absent	438	86,90	52	11,87	
Greater confidence in the hospital	Present	145	28,77	14	9,66	
	Absent	359	71,23	47	13,09	
Previous medical therapy without benefit	Present	50	9,92	10	20,00	
	Absent	454	90,08	51	11,23	
Reasons related to health services						
Too long time for booking exams	Present	99	19,64	20	20,20	*
	Absent	405	80,36	41	10,12	
ED has more tools to solve clinical problems	Present	205	40,67	21	10,24	
	Absent	299	59,33	40	13,38	
Easy accessibility of ED	Present	20	3,97	5	25,00	
	Absent	484	96,03	56	11,57	

**Table 5:** Distribution of non urgent ED visits according to clinical characteristics.

Variable		Total		Non urgent (tot 61)		^A^p-value
		N	%	N	%	
Presence of chronic disease	Present	198	39,29	13	6,57	
	Absent	256	50,79	40	15,63	*
	Missing	50	9,92	8	16,00	
Charlson Index Score	0 points	161	31,94	25	15,53	
	1–2 points	143	28,37	21	14,69	
	3–4 points	114	22,62	10	8,77	
	≥5 points	86	17,06	5	5,81	
Presence of chronic ongoing drug therapy	Present	163	32,34	9	5,52	
	Absent	259	51,39	39	15,06	*
	Missing	82	16,27	13	15,85	
Self perceived health status	Verygood	47	9,33	10	21,28	
	Good	186	36,90	23	12,37	
	Moderate	180	35,71	20	11,11	
	Poor	56	11,11	4	7,14	
	Verypoor	14	2,78	0	0,00	
	Missing	21	4,17	4	19,05	
Clinical examinations in the past 12 months	Present	341	67,66	40	11,73	
	Absent	147	29,17	21	14,29	
	Missing	16	3,17	0	0,00	
ED visits in the past 12 months	Present	218	43,25	31	14,22	
	Absent	269	53,37	25	9,29	*
	Missing	17	3,37	5	29,41	
ED arrival	Independent decision/by suggestion of relatives or friends	294	58,33	39	13,27	
	On medical advice	202	40,08	19	9,41	*
	Missing	8	1,59	3	37,50	
Attempting to contact the GP before arriving at the ED	Present	169	33,53	22	13,02	
	Absent	277	54,96	31	11,19	
	Missing	58	11,51	8	13,79	
Duration of the clinical problem	1 hour	89	17,66	1	1,12	
	Less than one day	141	27,98	6	4,26	*
	More than one day	263	52,18	54	20,53	
	Missing	11	2,18	0	0,00	

^a^p-Value

*<0.05

## Discussion

Hospital readmissions accounted for 6.39% of all the hospitalizations in the analysed sample, a smaller proportion than that (12.9%) registered in England [26], as well as those recorder in other settings, such as United States [27, 28], and Canada (8.0%) [29].The predictive model has shown that patient’s age, hospitalization in Geriatric care, some chronic diseases such as ischemic heart disease and chronic renal failure are the most important variables associated with an increased risk of readmission in the evaluated sample. In contrast with results from Halfon et al. [30], long length of hospital stay was not an independent predictor for hospital readmission. In fact, we found in our study that repeated admissions are more common within patients with previous early hospital discharge. In literature many models to predict risk of readmission have been evaluated; the main variables predicting readmission are male sex, length of stay, the presence of comorbidities, as well as some specific clinical conditions such as diabetes and chronic renal failure among others [26, 31, 32, 33].

Moreover, at a higher level, the multilevel analysis has shown that in the Marches Region, the local health authority plays an important role, highlighting the essential collaboration between the hospital and the extra-hospital level in ensuring continuity of care after discharge. The role of geographical, area level, has been reported also in different healthcare systems, [34] we believe that this associations maybe linked to the proximity of hospitals to the living address of patients, leading to an easy access of patients to the hospital setting, or to organizational, healthcare factors, grouping patients living in different areas; in particular, the organization of primary healthcare in Italy, strongly resides in the local district, and, in fact, this area level measure was capable of changing the role of socio-economic deprivation of the living area. Dealing with the ongoing efforts in improving the efficiency of the healthcare systems, and involving also the reduction of hospital beds, specific identification of patients at high risk may be useful in targeting interventions that can reduce the risk of readmission. In this context, besides the role of local district-level, primary care assistance, the 30-days readmission rate may be linked to hospital activity indicators, such as the volume of admissions per year, a factor that has been related to the increasing re-admission rate in the literature.[35] Besides the differences in general access, and organization of healthcare, the role of volumes in hospital activity are important in Italy too, where an historically inflated hospital based systems, besides the reduction in number of available hospital beds may still contribute to the presence of available beds to be occupied by chronic subjects. This area of study may be the more specific objective of further research.

On the other hand, the analysis of data relating to the utilization of the ED for non-urgent visits are partially in line to those found in similar Italian studies [25], where the odds of performing a non-urgent ED visit were significantly higher in younger and in patients with clinical problems of longer duration. Moreover, data have highlighted the poor utilization of primary care visits by young people, not affected by chronic conditions. Nevertheless, we have registered a considerable proportion (58.33%) of patients self-referring to the ED; this percentage of patients by-passing the GP visit is slightly lower than that recently registered in Dutch citizens (60.0%) [36].

One of the limitations of the manuscript may reside in the quite homogeneous residency of people visiting the ED of the main hospital of the Region; in fact, patients belonging to a limited number of local health districts have made the regional level analysis impossible for the ED visits. Moreover, for the above analysis, the DAI was assumed of being constant, across the study period, in each municipality of the region. The local-level indicators are made available on a 10-year basis by the Italian National Institute of statistics, this may be a limitation of the socio-economic evaluation, however, we may assume that a two-years window maybe considered quite stable for major socio-economic events. In the light of previous results, data have shown a double profile of people that do not find an appropriate answer in primary care in Central Italy. On one hand, we should work on ameliorating the continuity of care for the elderly, also by the early/automatic identification of patients at high risk of readmission, at the moment of patient hospital admission, and through the utilization of administrative data, too. On the other hand, the youngest seem to by-pass primary care physicians in order to receive a prompt high-tech, complex evaluation, and diagnosis, thus contributing to the inefficiency of the system. These results are in line from other evidences belonging to the recent literature, that has found various, complex reasons for patients to refer to the ED [37], and may support the findings related to the accessibility/convenience of ED as a valid reason for self-referral of patients [36]. Finally, those results may suggest the importance of a redesign of primary care in Italy. In fact, dealing with the particular reality of Italian healthcare system, we can register an important effort of primary care services in taking care of chronic patients, which is apparently not sustained by a prompt collaboration of the hospital setting, probably resulting in that little 2% of hospital stay ending with home care [38].

In this context, recent experiences have highlighted the potential role of specialized medical homes [39], and of personalized appointments in the ED [40], as a possible solution to this problem.

## Conclusions

The Italian primary care system, based on a universalistic access, is possibly suffering from the increasing age and comorbidity of citizens, and the increasing perception of diagnostic technologies as the sole offering an appropriate diagnosis, despite the pivotal role given to GP. In this context, the adoption of a recently proposed hospital-integrated general practice model may be beneficial [41]. In fact, a comprehensive approach, including improvement in health literacy of the young population, together with a novel design of the system with an improvement in the confidence in general practitioners, and the provision of basic diagnostic facilities may be important in order to improve ED utilization.

## Supporting Information

S1 Questionnaire (DOCX)
