# Supplementary material for: Emergency Department Non-Urgent Visits and Hospital Readmissions Are Associated with Different Socio-Economic Variables in Italy
Source: PLoS One. 2015 Jun 15;10(6):e0127823. doi: 10.1371/journal.pone.0127823 (PMC4468197; doi:10.1371/journal.pone.0127823)
Supplement: S1 Questionnaire — (DOCX) [file pone.0127823.s001.docx]

***Questionnaire***

***”*** Monitoring of ED utilization”

| SECTION A:PATIENT SOCIAL-DEMOGRAPHICAL FEATURES **Verb. PS** N°_______ | |
| --- | --- |
| 1. Marital status:   **①** Bachelor/Spinster  **②** Married  **③** Common law wife/husband  **④** Separated - Divorced  **⑤** Widow | 1. Qualification:   **①** None  **②** Elementary  **③** Secondary School  **④** High School  **⑤** Graduate and post-graduate |
| 1. Job:   Now you are:  **①** Student  **②** Unemployed  **③** Unfit for work  **④** Housewife  **⑤** Retired  If you work or you worked you can express your job condition ( If you are retired you can express your job before your retirement).  **⑥** Manager/Businessman / Freelancer  **⑦** Craftsman/Merchant/ Skilled Technician  **⑧** Employer/Teacher  **⑨** Worked | |
| 1. You live with:   **①** Alone  **②** With your family  **③** With a carer  **④** With friend or workmates  **⑤** With countrymen | 1. **Where do you live?**   **①** Property Home  **②** Rent Home  **③** Retirement home**/** Senior housing  **④** Community/ Day care  **⑤** Other ______________________ |
| 1. Ticket Exemption for:   **①** Pathology or disability  **②** Income  **③** Age  **④** Pregnancy | 1. **How far is the hospital from do you live?**   **①** Less than 5 km  **②** 6-35 km  **③** More than 35 km |

| SECTION B: DESCRIPTION AND REASONS **FOR THE ADMISSION TO THE FIRST AID** |
| --- |
| 1. Did you accomplished any health examinations in the last 12 months? (only one answer)   **①** Yes  **②** Not |
| 1. Did you go to the First Aid in the last 12 months? (only one answer)   **①** Yes  **②** Not |
| 1. Do you know your family doctor’s consulting time? (only one answer)   **①** Yes  **②** Not |
| 1. Does your family doctor work with other doctors? (in medical fraternity)? (only one answer)   **①** Yes **②**Not **③**Don’t know   1. Do you know the telephone number or the address of the emergency medical service that works every night and during the weekends?(only one answer)   **①**  Yes  **②** Not |
| 1. **If you are a foreign patient and you are not recorded in a family doctor’s list,** ( at the contrary go to answer number 14)**, do you know the doctor’s office for foreign patients(STP)?** (only one answer)   **①** Yes  **②** Not |
| 1. You are arrived at First Aid on recommendation of: (only one answer)   **①** Family doctor **④** Own decision **⑦** Sent by cup  **②** Emergency medical service **⑤** Relatives/Friends **⑧** Moved from an institute  **③** Hospital specialist **⑥** Pharmacist |
| 1. **If you have been sent by a doctor specify:** (only one answer)   **①** After a medical visit  **②** After a call  **③** With your family doctor’s priority request |
| 1. Did you look for your family doctor before going to the First Aid? (only one answer)   **①** Yes  **②** Not  **③** I haven’t got the family’s doctor |
| 1. Why do you come to the First Aid? (More answers)   **17a. Personal reasons**  **①** I thought there weren’t time/ I scared for a symptom  **②** I’m very busy at work and I have no time to go to my family doctor or to a specialist during the consulting time  **③** I have economic problems to pay the ticket for the required exams  **④** I have just had a traumatic accident  **⑤** I’m on holiday or for work/ I’m a student and I haven’t got a family doctor  **⑥** I need a medical certificate because of my work  **⑦** My pharmacist’s suggestion doesn’t satisfied me  **⑧** I come right from my work place |
| **17b. Reasons linked to my family doctor**  **①** My family doctor’s treatment has been ineffectual  **②** There was a large queue at my family doctor  **③** There was the doctor substitute  **④** I had many difficulties to have a date with my family doctor  **⑤** Doctor’s office closed  **⑥** I trust in hospital because the specialists have more knowledge than my family doctor |
| **17c. Reasons linked to Health Services:**  **①** Reservation time are too long  **②** Home care (ADI) is not active on Sundays and public holidays  **③** I thought the First Aid had more instruments to resolve my problems/ had a short time to solve the problem  **④**  It’s the nearest place / easily available and it’s always open  **⑤** Because it’s free  **⑥** None of this reasons |

| SECTION C. PERCEPTION ABOUT YOUR STATE OF HEALTH **Verb. PS** N°_______ | |
| --- | --- |
| 1. How long does it last your problem? (only one answer)   **①** 1 Hour  **②** Less than 24 hours  **③** More than 24 hours  **④** A week  **⑤** 2 weeks  **⑥** More than 1 month | |
| 1. **Would you say that in general your health is?** (only one answer)   **①** Excellent  **②** Very good  **③** Good  **④** Bad  **⑤** Very bad   1. Now thinking about your physical health, which includes physical illness and injury, for how many days during the past 30 days was your physical health not good?   **N°___** days   1. Now thinking about your mental health, which includes stress, depression, and problems with emotions, for how many days during the past 30 days was your mental health not good?   **N°___** days   1. During the past 30 days, for about how many days did poor physical or mental health keep you from doing your usual activities, such as self-care, work, or recreation?   **N°___** days | |
| 1. Do you suffer chronic diseases?   **①** Not **②** Yes | |
| 1. **If yes, which ones**? | |
| 1. 🞅 Myocardial infarct 2. 🞅 Congestive heart failure 3. 🞅 Peripheral vascular disease (arteriopathy lower limbs) 4. 🞅 Cerebro-vascular disease (Ictus) 5. 🞅 Dementia 6. 🞅 Chronic lung disease (bronchitis, asthma) 7. 🞅 Connective tissue disease (arthritis, arthrosis) 8. 🞅 Ulcer 9. 🞅 Chronic liver disease (liver enlarged) 10. 🞅 Diabetes | 1. 🞅 Hemiplegia (paralysis) 2. 🞅 Chronic liver disease 3. 🞅 Diabetes with complication 4. 🞅 Tumour 5. 🞅 Leukaemia 6. 🞅 Lymphoma 7. 🞅 Moderate or severe kidney disease (cirrhosis, hepatitis) 8. 🞅 Malignant tumour with metastasis 9. 🞅 AIDS   🞅 Other___________________ |
| 1. **Do you take drugs to keep under control any chronic disease?**   **①** Yes **②** Not | |

***The interview is ended***

***Thanks for your collaboration!***
